# Supplementary material for: Stochastic modeling of aging cells reveals how damage accumulation, repair, and cell-division asymmetry affect clonal senescence and population fitness
Source: BMC Bioinformatics. 2019 Jul 15;20:391. doi: 10.1186/s12859-019-2921-3 (PMC6631810; doi:10.1186/s12859-019-2921-3)
Supplement: Supplementary file 1 — Figure S1. A-H. Relative representation of the other parameters in the cases where changing the level of inheritance caused a significant (> 5%) fitness difference. Figure S2. A-H. Relative representation of the other parameters in the cases where changing the maximum repair rate caused a significant (> 5%) fitness difference. Figure S3. A-H. Relative representation of the other parameters in the cases where changing the Michaelis constant km for repair caused a significant (> 5%) fitness difference. Figure S4. A-H. Relative representation of the other parameters in the cases where changing the damage accumulation rate caused a significant (> 5%) fitness difference. Table S1. Parameters with fixed mean values. Table S2. Parameters with values selected from a grid of values. (PDF 1035 kb) [file 12859_2019_2921_MOESM1_ESM.pdf]

Stochastic modeling of aging cells reveals how damage accumulation, repair, and cell-division asymmetry affect clonal senescence and population fitness

Ruijie Song[1,2] and Murat Acar[1,2,3,4,*]

[1] Systems Biology Institute, Yale University
850 West Campus Drive, West Haven, CT 06516

[2] Interdepartmental Program in Computational Biology and Bioinformatics, Yale University
300 George Street, Suite 501, New Haven, CT 06511

[3] Department of Molecular Cellular and Developmental Biology, Yale University
219 Prospect Street, New Haven, CT 06511

[4] Department of Physics, Yale University
217 Prospect Street, New Haven, CT 06511

* To whom correspondence should be addressed: murat.acar@yale.edu

SUPPLEMENTARY INFORMATION

Supplementary Figures S1-S4

Supplementary Tables S1-S2

SUPPLEMENTARY FIGURES

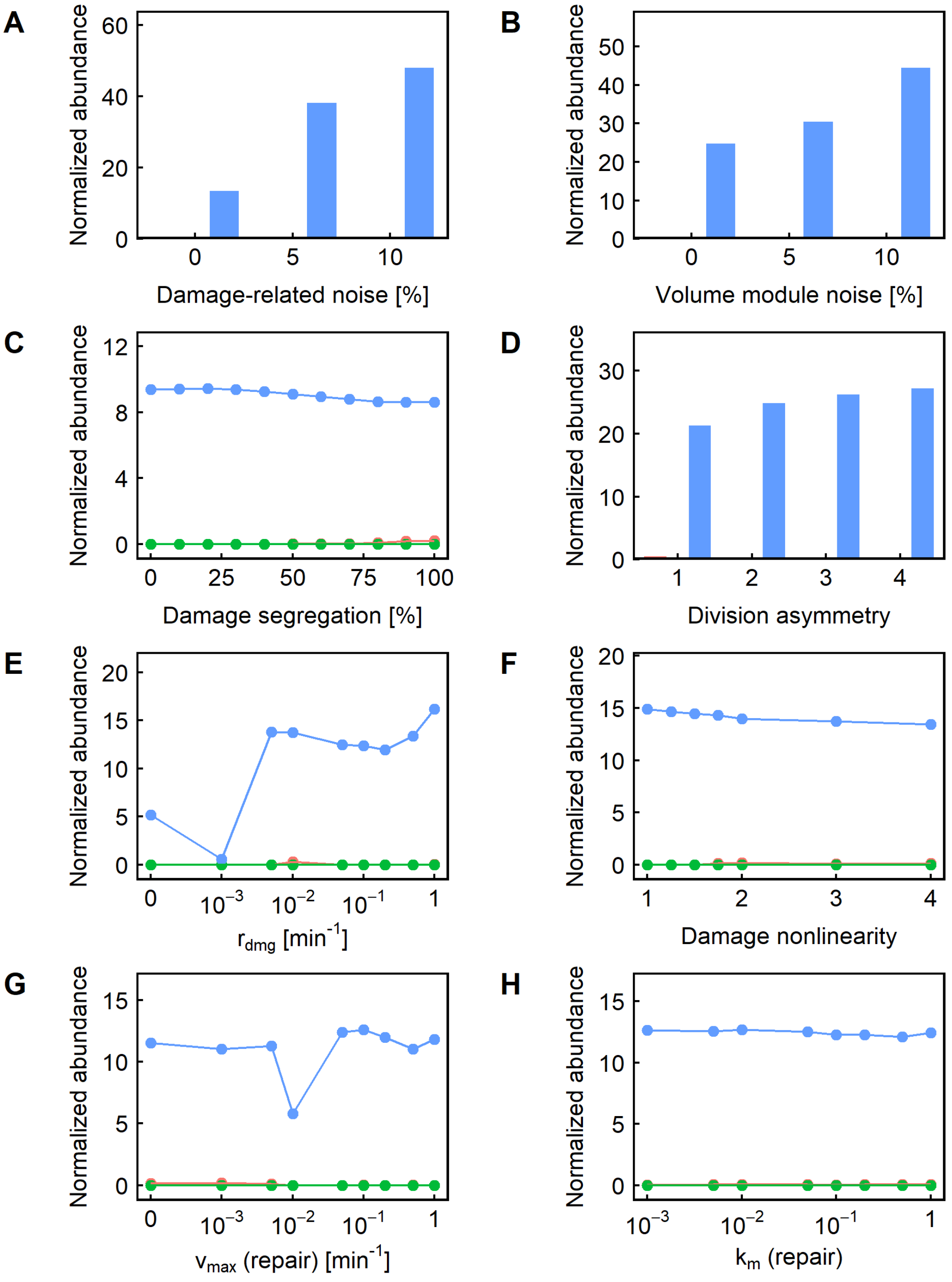

Fig. S1. A-H. Relative representation of the other parameters in the cases where changing the level of inheritance caused a significant (>5%) fitness difference. In each panel, the color indicates the level of inheritance resulting in maximum fitness: red indicates that no inheritance (c = 0%) is the most fit; blue indicates that maximum inheritance (c = 90%) is the most fit; and green indicates that an intermediate level of inheritance is the most fit.

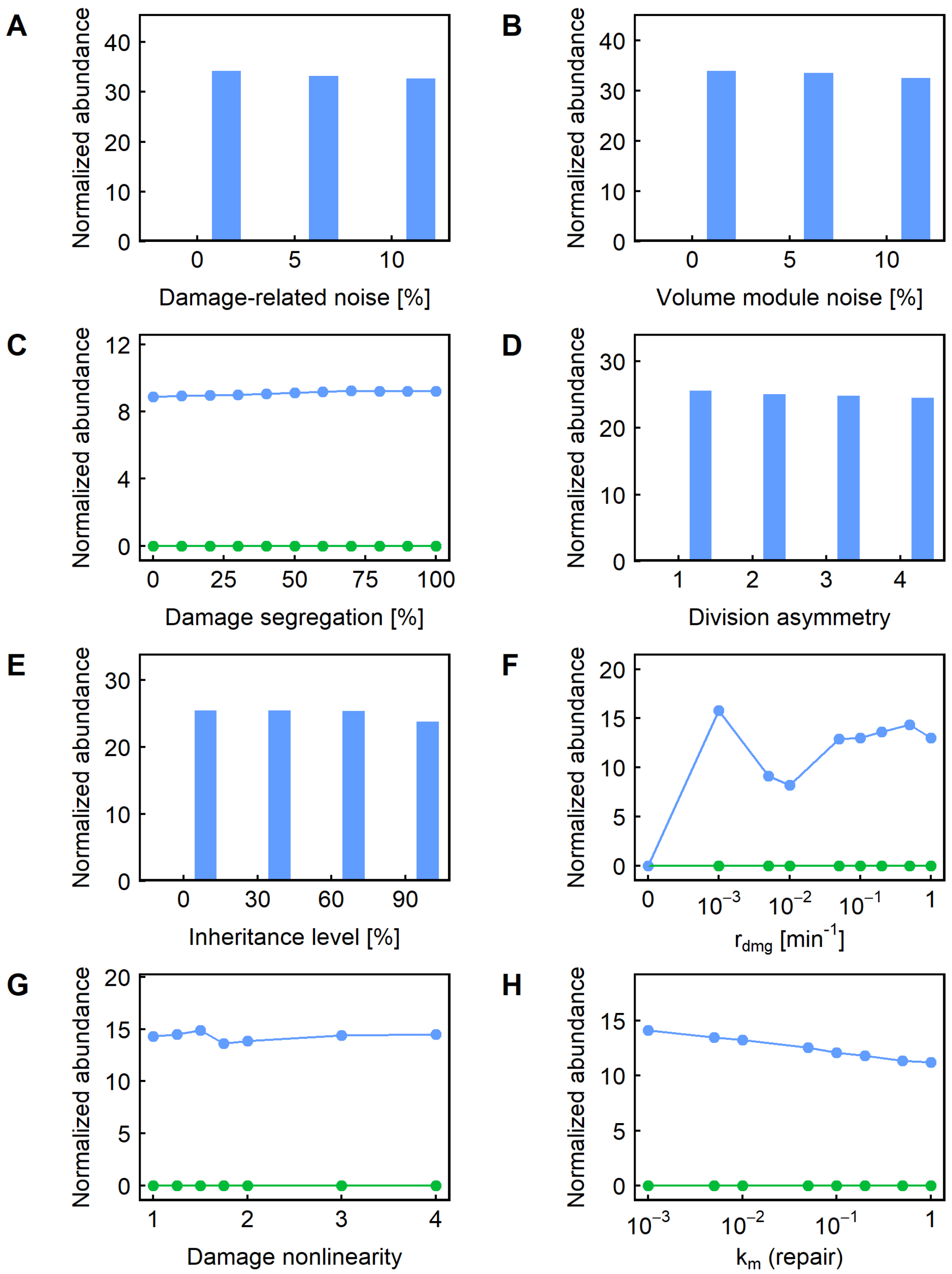

Fig. S2. A-H. Relative representation of the other parameters in the cases where changing the maximum repair rate caused a significant (>5%) fitness difference. In each panel, the color indicates the level of repair resulting in maximum fitness: red indicates that no repair is the most fit; blue indicates that maximum repair ($v_{max} = 1\,\text{min}^{-1}$) is the most fit; and green indicates that an intermediate rate of repair is the most fit.

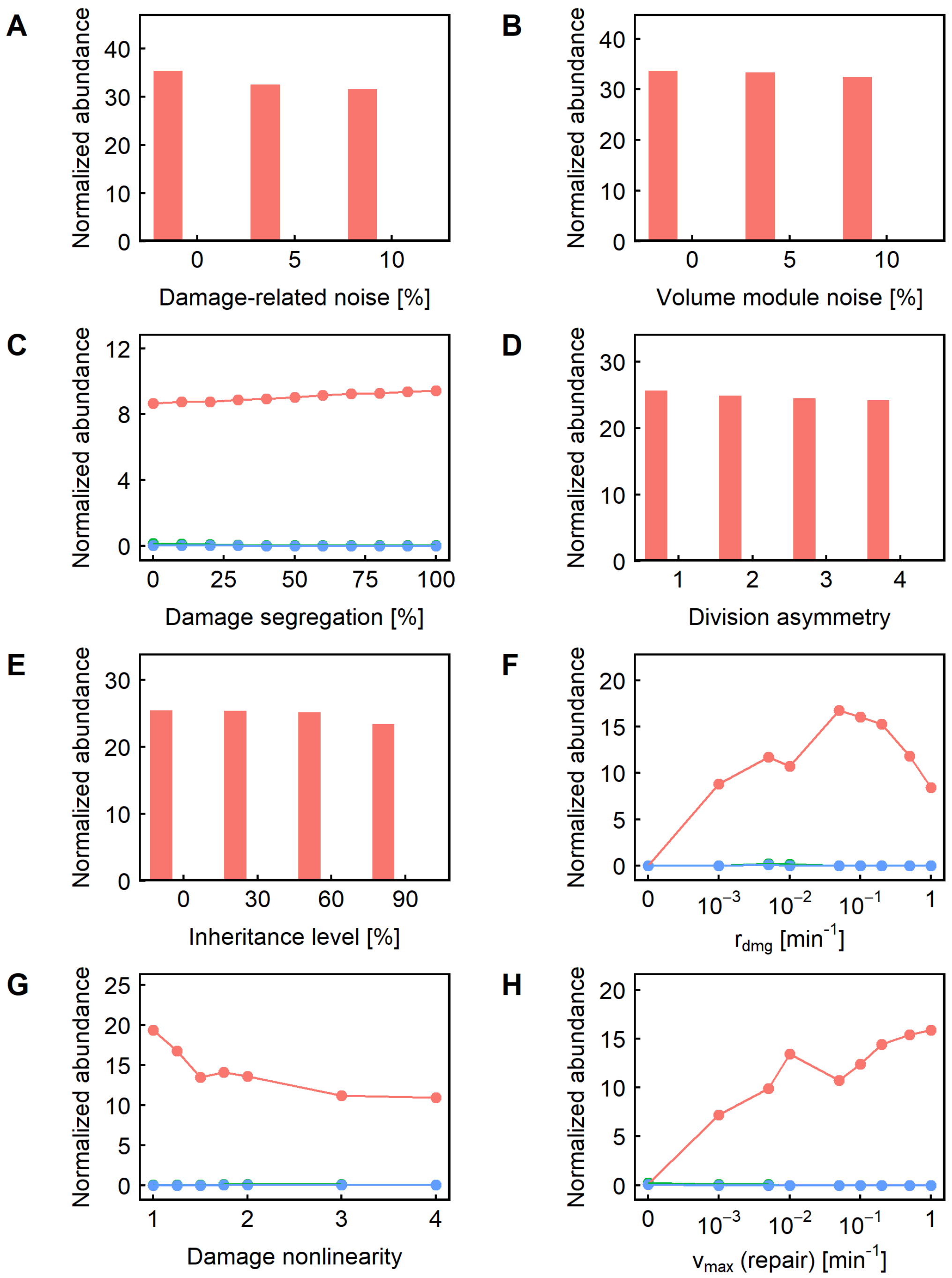

Fig. S3. A-H. Relative representation of the other parameters in the cases where changing the Michaelis constant k_m for repair caused a significant (>5%) fitness difference. In each panel, the color indicates the value of k_m resulting in maximum fitness: red indicates that the minimum value $k_m = 0.001$ is the most fit; blue indicates that maximum k_m ($k_m = 1$) is the most fit; and green indicates that an intermediate value of k_m is the most fit.

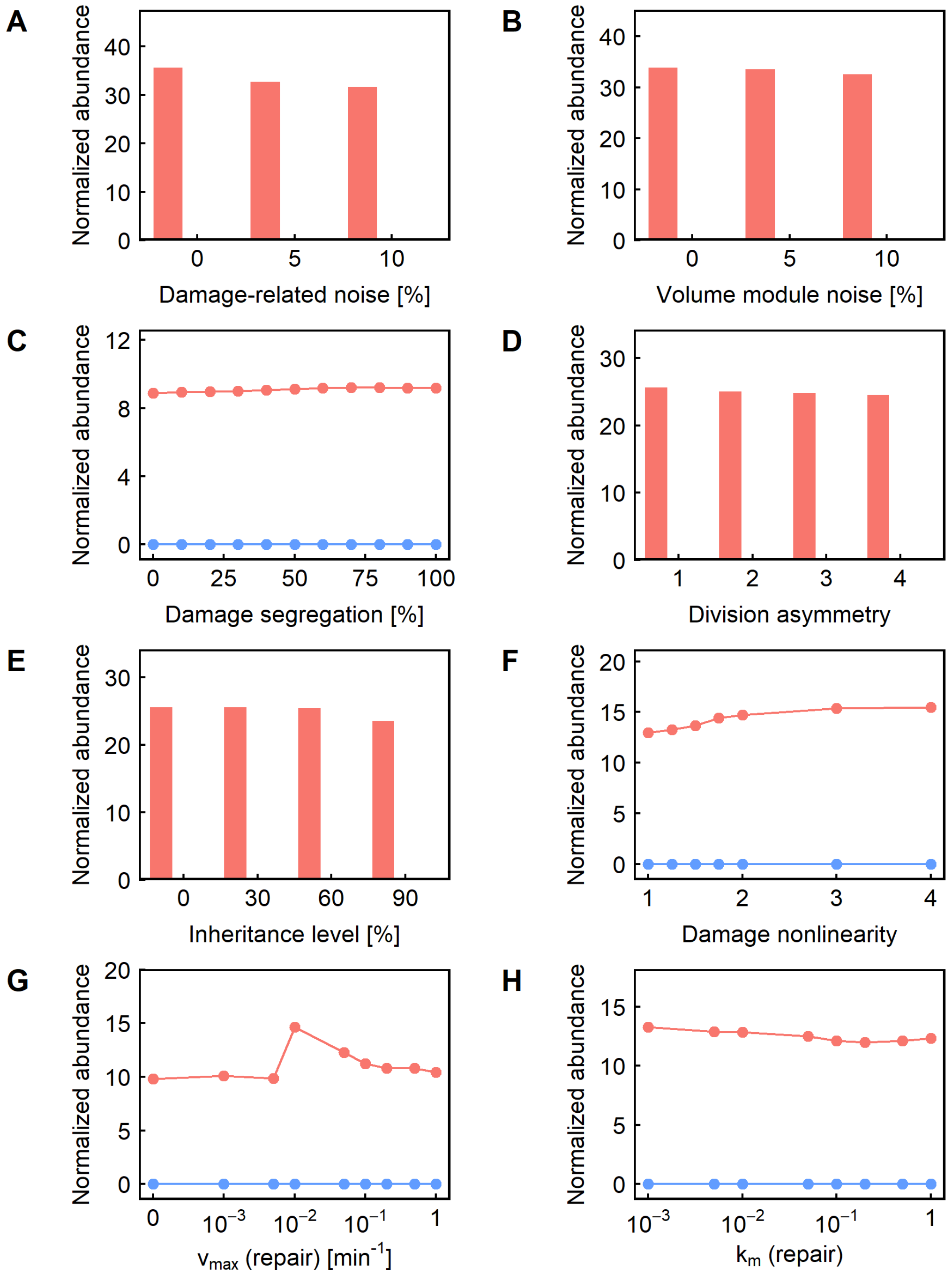

Fig. S4. A-H. Relative representation of the other parameters in the cases where changing the damage accumulation rate caused a significant (>5%) fitness difference. In each panel, the color indicates the damage accumulation rate resulting in maximum fitness: red indicates that no damage accumulation is the most fit; blue indicates that maximum damage accumulation ($r_{dmg} = 1\,\text{min}^{-1}$) is the most fit; and green indicates that an intermediate rate of damage accumulation is the most fit.

SUPPLEMENTARY TABLES

Parameter	Meaning	Value	Unit
r_{growth}	Volume growth rate constant	0.0072	min^{-1}
V_i	Initial volume	50	fL
V_{crit}	Critical volume (as function of generation *g*)	106.7 + 4.628*g*	fL

Table S1. Parameters with fixed mean values.

Parameter	Meaning	Values	Unit
c	Level of inheritance of parameter values	0, 30, 60, 90	%
R	Mother/daughter volume ratio at division	1, 2, 3, 4	
n_d	Damage-related noise	0, 5, 10	%
n_v	Volume module noise	0, 5, 10	%
s	Level of damage segregation	0, 10, 20, 30, 40, 50, 60, 70, 80, 90, 100	%
v_{max}	Maximum rate of repair	0, 0.001, 0.005, 0.01, 0.05, 0.1, 0.2, 0.5, 1	min^{-1}
k_m	Michelis constant for repair	0.001, 0.005, 0.01, 0.05, 0.1, 0.2, 0.5, 1	
r_{dmg}	Rate of damage accumulation	0, 0.001, 0.005, 0.01, 0.05, 0.1, 0.2, 0.5, 1	min^{-1}
α	Nonlinearity of the effect of damage on growth rate	1, 1.25, 1.5, 1.75, 2, 3, 4	

Table S2. Parameters with values selected from a grid of values.
